# STUDY OF ROLE OF STREPTOCOCCAL THROAT INFECTION IN PITYRIASIS ROSEA

**DOI:** 10.4103/0019-5154.44787

**Published:** 2008

**Authors:** Madhuri Parija, Devinder Mohan Thappa

**Affiliations:** *From the Department of Dermatology and STD, Jawaharlal Institute of Postgraduate Medical Education and Research (JIPMER), Pondicherry - 605 006, India*

**Keywords:** *Pityriasis rosea*, *streptococcal throat infection*, *ASLO titer*

## Abstract

**Background:**

Pityriasis rosea is a common, acute exanthem of uncertain etiology. The exact cause of pityriasis rosea is not known but various hypotheses have been postulated incriminating infective agents such as viruses, bacteria, spirochete and noninfective etiologies such as atopy and autoimmune causes have also been investigated.

**Aim:**

We undertook a study to investigate the role of *Streptococcus haemolyticus* in the causation of pityriasis rosea and study the levels of C-reactive protein (CRP) and ASLO titer in patients with pityriasis rosea.

**Materials and Methods:**

The study included 20 patients with pityriasis rosea attending the outpatient dermatology department at JIPMER hospital during the period from June to December 2004. Corresponding number of age- and sex-matched controls were chosen from amongst healthy individuals and patients attending skin OPD with dermatological disorders other than pityriasis rosea.

**Results:**

On analyzing the data collected from 20 cases of pityriasis rosea, the average age was found to be 15.3 years and ranged from 5 years to 30 years. The male to female ratio was found to be 1.5:1. The average duration of illness was 14.5 days (median) and 29.3 days (mean). CRP was negative in all the cases as well as the controls. ASLO titer was found to be raised in 2 (10%) cases, while it remained below the critical value in all the controls. On comparing the cases and controls, the raised ASLO titer in the cases was found to be not statistically significant (p = 0.147). From the throat swab culture, *Streptococcus haemolyticus* was isolated from only one case and none of the controls. This finding was not statistically significant (p = 0.310).

**Conclusion:**

As per the findings of the present study, we arrived at conclusion that there is no association between streptococcus pharyngitis and pityriasis rosea.

## Introduction

Pityriasis rosea is a common, acute exanthem of uncertain etiology.^1^ Viral and bacterial causes have been sought, but convincing answers have not yet been found. This self-limiting, scaly, papulosquamous skin eruption accounts for approximately 1% of dermatoses observed in dermatology office.^2^ Its distinguishing clinical characteristics are the presence of an initial red scaling patch (herald patch) followed 7–14 days by a more generalized eruption of red oval patches on the non-sun-exposed areas of the body. The disease is frequently asymptomatic, although pruritus may be present in few patients. Pityriasis rosea can occur throughout the year, but more commonly is observed during the winter, spring and autumn months. The age group commonly afflicted with this disease is children and young adults (10–35 years) with a peak between 10 and 14 years. Female to male ratio is approximately equal,^3^ whereas in another study, it has been found to be 1.5:1.^4^ The lesions are distributed mainly on the trunk and proximal limbs; however, the secondary skin eruptions may also present on the face and extremities. Mucous membrane lesions occur in 16% of all pityriasis rosea cases, but due to lack of symptoms, these oral lesions are often overlooked and are rarely recorded.^5^–^8^ The exact cause of pityriasis rosea is not known, but various hypotheses have been postulated; incriminating infective agents such as viruses, bacteria, spirochete and noninfective etiologies such as atopy and autoimmune causes have also been investigated.^3^,^4^ We undertook a study to investigate the role of *Streptococcus haemolyticus* in the causation of pityriasis rosea and study the levels of C-reactive protein (CRP) and ASLO titer in patients with pityriasis rosea.

## Materials and Methods

The study included 20 patients with pityriasis rosea attending the outpatient dermatology department at JIPMER hospital during the period from June to December 2004. The diagnosis of pityriasis rosea was clinically made on the basis of the detailed history and typical examination findings. Cases with positive venereal disease research laboratory (VDRL) test and patients suspected of having other disorders such as fungal infection, secondary syphilis, eczema or psoriasis were excluded from the study. Corresponding number of age- and sex-matched controls were selected from amongst healthy individuals and patients who attended the skin OPD with dermatological disorders other than pityriasis rosea. An informed consent was taken from each patient or accompanying guardian for inclusion in the study.

Patients with pityriasis rosea were subjected to a detailed clinical history and a thorough examination of the skin, mucous membrane, hair and nail. Age, sex, occupation, place of residence, duration of illness, history of preceding upper respiratory tract infection and family history, site of lesion, morphology of skin lesions, involvement of hair, nail, mucous membrane, etc., were recorded in a proforma. KOH mount of skin scrapings was performed to rule out tinea. Blood samples (5 ml) and throat swabs were collected from the cases and controls. Serum extracted from the blood was subjected to C-reactive protein (CRP) and ASLO estimation by standard serological procedures. VDRL test was performed on the sera collected from the cases. Throat swabs were sent for bacteriological examination and routine culture and sensitivity for aerobic bacteria. Statistical analysis was performed by means of chi-square test for differences in proportion and student *t* test for differences in means.

## Results

On analyzing the data collected from 20 cases of pityriasis rosea, the average age was found to be 15.3 years and ranged from 5 years to 30 years. The male to female ratio was found to be 1.5:1 (Table 1). The average duration of illness was 14.5 days (median) and 29.3 days (mean). Since the mean was unduly influenced by few extreme values in the data, the median was taken to be more reliable. Eight (40%) out of 20 patients gave a history of upper respiratory tract infection preceding the appearance of skin rash. None of them gave history of similar complaints in the family. Skin lesions were maximally found on the trunk (95%) followed by limbs (45%) and face (40%). Hair, nail and mucous membrane were not involved in any of the cases. All cases had classical pityriasis rosea lesions, except one that was a case of inverse pityriasis rosea.

**Table 1:** Baseline characteristics of the cases and controls

Parameters	Pityriasis rosea group	Control group	*p* value
Mean age (years)	15.25 (±6.93)	14.85(±7.39)	0.861
Male / female	1.5:1	1.5:1	--

CRP was negative in all the cases as well as controls (Table 2). ASLO titre was found to be raised in 2 (10%) cases, while it remained below the critical value in all the controls. On comparing the cases and controls, the raised ASLO titer in the cases was found to be statistically insignificant (*p* =0.147).

**Table 2:** Comparison of raised ASLO titer between cases of pityriasis rosea and controls

Parameter	Pityriasis rosea group no. (%)	Control group no. (%)	*p* value
Raised ASLO titre (≥200 IU/ml)	2 (10)	0 (0)	0.147

From throat swab culture,*Streptococcus haemolyticus* was isolated from only one case and none of the controls (Table 3). This finding was not statistically significant (*p* =0.310).

**Table 3:** Comparison of throat swab culture reports between cases of pityriasis rosea and controls

Parameter	Pityriasis rosea group no. (%)	Control group no. (%)	*p* value*
*Streptococcus haemolyticus*	1 (5)	0	0.310
Normal flora			
	14 (70)	17 (85)	--
*Staphylococcus aureus*	2 (10)	0	--
Contamination			
	3 (15)	3 (15)	--

* when compared with the normal flora and *Staphylococcus aureus* group

## Discussion

Pityriasis rosea, first named as such in 1860, possibly holds the longest record for an exanthem suspected to be associated with an infection; however, an exact cause for this has not been found.^9^ The distinctly programmed clinical course, the lack of recurrence for most patients, and the presence of temporal case clustering provide the strongest evidence to support an infectious etiology. Further support comes from seasonal variation and the association with respiratory tract infections, the unfavorable social and economic background of cases, and a history in some cases of contact with patients with pityriasis rosea. The apparent therapeutic efficacy of several treatment modalities does not provide strong evidence for or against an infectious etiology. The roles of human herpesvirus 7 and to a lesser extent human herpesvirus 6 remain controversial. There exists reasonable evidence that pityriasis rosea is not associated with cytomegalovirus, Epstein-Barr virus, parvovirus B19, picornavirus, influenza and parainfluenza viruses, Legionella spp., Mycoplasma spp. and Chlamydia spp. infections. Evidence is also unsubstantiated as yet for alternative etiological hypotheses such as autoimmunity, atopy, and genetic predisposition.^9^ As per the recent review, there is insufficient evidence that human herpesvirus 7 infection is causally related to pityriasis rosea.^10^

Early publications attributed it to the wearing of unwashed clothes, insect bite and even to a psychogenic conditions.^11^ However, the natural history and rarity of second attacks have given rise to the belief that it is an infectious disease.^12^ Various microorganisms including fungi, spirochetes, streptococci and viruses^5^,^13^,^14^ have been implicated in many studies.^3^,^4^ The epidemiological evidence for a possible infective etiology consists of the following: first, seasonal variation in incidence; second, the occurrence of mild prodromal symptoms; third, the strong association with recent upper respiratory tract infection preceding the cutaneous rash in pityriasis rosea^15^ and fourth, a high incidence among people clustered together in educational institutes or within families,^12^,^16^ e.g., dermatologists who are often in contact with pityriasis patients are affected 3–4 times compared to other doctors.^17^ Sharma *et al*.^4^ in their study of erythromycin in pityriasis rosea suggested that an infectious agent sensitive to erythromycin may cause pityriasis rosea and in their study raised antistreptolysin O titers were found in 34 of the total 90 patients (37.7%), thereby suggesting the involvement of Streptococcus in pityriasis rosea. Since a few studies suggest indirectly that although Streptococcus is a probable culprit, very few have attempted to directly show a relation between Streptococcus and pityriasis rosea, hence this study.

Thus, various studies have shown the role of infective agents in causation of pityriasis rosea,^3^–^5^,^12^–^14^ whereas a few studies have found fungi, streptococci, mycoplasma and spirochetes to be unrelated to the pathogenesis of pityriasis rosea.^18^ The objective of the present study is to find an association between streptococcal pharyngitis and pityriasis rosea. The present study showed an almost equal involvement of males and female (male/female ratio = 1.5:1).This is in concurrence to the earlier reports in which an equal preponderance or female preponderance has been reported.^15^,^19^,^20^

Preceding history of upper respiratory tract infections were recorded in 8 out of 20 cases (40%), which suggests a possible infective etiology. However, the percentage of cases giving a positive history was found to be less compared to the percentage (68.88%) reported by Sharma *et al*.^4^ Raised acute phase reactants in cases of pityriasis rosea reported by Sharma *et al*.^4^ have not been found in the present study. There was no rise in CRP level and no significant rise in ASLO titer in the cases. *Streptococcus haemolyticus* was isolated from only one case and hence is insignificant.

As per the findings of the present study, we have arrived at a conclusion that there is no association between streptococcus pharyngitis and pityriasis rosea. Hence, prescribing antibiotics to patient is completely unnecessary and undesirable. However, in the absence of a large sample, the present study cannot be taken as a final answer to role of *Streptococcus haemolyticus* in the causation of pityriasis rosea.
